# Health promotion programme design and efficacy in relation to ageing persons with culturally and linguistically diverse backgrounds: a systematic literature review and meta-analysis

**DOI:** 10.1186/s12913-015-1222-4

**Published:** 2015-12-16

**Authors:** Qarin Lood, Greta Häggblom-Kronlöf, Synneve Dahlin-Ivanoff

**Affiliations:** Institute of Neuroscience and Physiology, Section for Health and Rehabilitation, The Sahlgrenska Academy, University of Gothenburg, Box 455, SE-405 30 Gothenburg, Sweden; University of Gothenburg Centre for Person-Centred Care (GPCC), Gothenburg, Sweden; University of Gothenburg Centre for Ageing and Health (Agecap), Gothenburg, Sweden

**Keywords:** Aged, Emigrants and immigrants, Global health, Healthcare disparities, Minority health

## Abstract

**Background:**

Health promotion has the potential to empower people to develop or maintain healthy lifestyles. However, previous research has visualised serious health and healthcare inequities associated with ageing, cultural affiliations and linguistic preferences. Therefore, this study was part of a larger health promotion project, set out to bridge barriers to health for ageing persons who have migrated to Sweden. More specifically, the present study aimed to elucidate the content and effects of multidimensional health promotion programmes in the context of ageing persons with culturally and linguistically diverse backgrounds.

**Methods:**

Databases were systematically searched to identify relevant randomised controlled trial publications. All potentially relevant publications were assessed for relevance and design and after this screening, a final sample of eight publications could be included in the review. Those publications evaluated six different programmes and a mixed-methods approach to data analysis was applied, using a combination of narrative synthesis, meta-analyses and evidence grading.

**Results:**

The findings suggest a multidimensional health promotion programme design for ageing persons with culturally and linguistically diverse backgrounds, involving culturally and linguistically modified activities and health information that should be provided by professionals with a person-centred approach. In addition, the meta-analyses revealed statistically significant effects in favour of health promotion on: general health, depression, mental health, physical health, and vitality. However, the evidence for the identified effects is low, and further research findings are likely to change the estimations.

**Conclusions:**

The present study provides an aggregation of health promotion intervention research with older persons with culturally and linguistically diverse backgrounds; a group of people who are commonly excluded from research, and marginalised when it comes to health and healthcare. By visualising the core components of health promotion programmes with proven efficacy, the findings provide guidance for further explorations of how health promotion should be designed to minimise inequities in health.

**Electronic supplementary material:**

The online version of this article (doi:10.1186/s12913-015-1222-4) contains supplementary material, which is available to authorized users.

## Background

Health promotion has become a fundamental component of healthcare services aimed at older persons [1], and it has been well documented that multidimensional health promotion programmes commenced before the onset of poor health can have a large impact on their possibilities to remain living independently their own homes [2–4]. According to the World Health Organization (WHO) [5], health promotion involves structured and multicomponent actions to provide equal opportunities for the whole population to take control over their health [5]. However, previous research has visualised serious inequities with regard to the opportunities of older persons with culturally and linguistically diverse (CALD) backgrounds to achieve their fullest health potential. Poor physical and mental health among older persons with CALD backgrounds has been frequently reported in the scientific literature [6–9]. Along with documentation on cultural and linguistic barriers to healthcare access [10–12], and health promotive behaviours [13], this calls for scientific explorations on what actions to undertake in order to promote the health of an ageing and increasingly diverse population.

With increased globalisation and survival rates, confronting barriers to health for older persons with different cultural, linguistic or national backgrounds than the majority population of the country they reside in is important from a public health, as well as an ethical perspective. Access to health should be considered a human right, not to be compromised by socioeconomic or cultural factors [14, 15]. Therefore, this study is part of a larger research project with the aim of implementing a health promotion programme for ageing persons who have migrated to Sweden [16]. The aim of the present study was to systematically review randomised controlled trial (RCT) publications in order to elucidate the content and effects of multidimensional health promotion programmes that have included ageing persons with CALD backgrounds. With focus on promoting the health of all people, regardless of cultural, linguistic or national affiliation, the authors addressed the following research questions:

1. Are there any commonalities among multidimensional health promotion programmes that have included ageing persons with CALD backgrounds in the study population?
2. Are there any documented effects of multidimensional health promotion programmes on the general, mental and physical health of ageing persons with CALD backgrounds?

## Methods

Cochrane [17], and PRISMA [18] guidelines were implemented to standardise the features of this systematic literature review. The PRISMA 2009 checklist is provided in Additional file 1.

### Selection criteria

All included publications were screened using the following criteria as filter: 1) randomised controlled trial design, 2) participants described as ageing, older or elderly, representing a diversity of cultural, linguistic, ethnic, or national backgrounds, 3) evaluating effects of multidimensional health promotion programmes on general health, physical health and mental health. Publications were excluded if the were not written in English. Only peer-reviewed publications were included since they are commonly considered to represent the highest quality literature. No limitations with regard to year of publication were applied.

For the meta-analysis, publications were selected based on the following eligibility criteria: 1) reporting between-group differences, 2) similarity across the study arms. Publications were excluded if they reported follow-up data of another, already included, trial.

### Study selection

A database search was carried out between October 2010 and February 2014 in the following databases: Amed, Cinahl, Cochrane controlled trials register, PubMed and Scopus. Assisted by a university librarian and other researchers in the field, the authors constructed chains of key words for each database in order to systematically search the variegated research field. All fields/text were searched unless indicated otherwise.

#### Pubmed search terms

((“culturally and linguistically diverse” OR “CALD” OR “culturally diverse” OR foreign-born” OR immigrants OR emigrants OR “immigrants and emigrants [MESH]) AND “health promotion”, (limit randomized controlled trial)); (“health promotion” [MESH] AND (intervention OR “health services for the aged” [MESH]), (limit randomized controlled trial)); ((“emigration and immigration [MESH]) OR “ethnic minority groups” OR “minority groups” OR “ethnically diverse” OR acculturation OR “cultural competence” OR “cultural congruence” OR “cultural sensitivity” OR acculturation OR multicultural OR bicultural OR intercultural OR transcultural OR “lifestyle redesign” OR “preventive home visits”, (limit randomized controlled trial)); ((“Ethnic groups” [MESH] AND “health promotion” [MESH], (limit randomized controlled trial)); ((“lifestyle intervention” OR diversity OR “lifestyle intervention”) AND health, (limit randomized controlled trial));

#### Scopus search terms

((“culturally and linguistically diverse” OR “CALD” OR “culturally diverse” [abstract, title, keyword]) AND “health promotion” [abstract, title, keyword])); (“culturally and linguistically diverse” OR “CALD” OR “culturally diverse” OR “linguistcally diverse” OR “foreign-born” OR “born abroad” [abstract, title, keyword]) AND (health AND intervention [abstract, title, keyword]); (“born abroad” AND health [abstract, title, keyword]); ((immigrants OR emigrants [abstract, title, keyword]) AND (health AND intervention AND randomized [abstract, title, keyword])); ((emigration OR immigration OR emigrants OR immigrants” OR “ethnic minority groups” OR “minority groups” OR ethnic groups”) AND randomized AND “health promotion”); (“lifestyle redesign”); ((immigration OR immigrants OR emigration OR emigrants OR “ethnic minority groups” OR “ethnic groups”) AND health AND randomized); ((“lifestyle intervention” AND health AND randomized) NOT diabetes); (acculturation AND randomized); ((diversity OR “cultural competence” OR “cultural congruence” OR “cultural sensitivity” OR multicultural OR bicultural OR intercultural OR transcultural) AND randomized AND health)

#### AMED search terms

(“culturally and linguistically diverse” OR “culturally diverse” OR “linguistically diverse” OR “CALD” OR “foreign-born” OR “born abroad” OR immigrants OR emigrants OR emigration OR immigration OR “ethnic minority groups” OR “minority groups” OR acculturation OR “cultural competence” OR “cultural congruence” OR “cultural sensitivity” OR multicultural OR bicultural OR intercultural OR transcultural OR “lifestyle redesign” OR “lifestyle intervention” OR “preventive home visits”); (“ethnic groups” AND health (limit clinical trial)); (diversity AND health [abstract])

#### CINAHL search terms

((“culturally and linguistically diverse” OR “linguistically diverse” OR “CALD” OR “lifestyle intervention” OR intercultural AND health [abstract]); (“culturally diverse” AND randomized AND health [abstract]); (“born abroad” OR emigrants OR “lifestyle redesign” OR “cultural congruence”); ((“foreign-born” OR “immigrants” OR “ethnic groups” OR “minority groups” OR emigration OR immigration OR transcultural OR bicultural OR multicultural OR “cultural sensitivity” OR “cultural competence” OR diversity OR acculturation) AND (randomized OR “randomized controlled trial”))

#### Cochrane clinical trials search terms

(“culturally and linguistically diverse” OR “culturally diverse” OR “linguistically diverse” OR “CALD” OR foreign-born” OR “born abroad” OR immigrants OR emigrants OR “immigrants and emigrants [MESH] OR “ethnic minority groups” OR “preventive home visits” OR “lifestyle redesign” OR “cultural competeonce” OR “cultural congruence” OR multicultural OR “cultural sensitivity” OR intercultural OR transcultural OR bicultural OR “preventive home visits” OR “lifestyle redesign”); ((“minority groups” OR “ethnic groups”) AND health); (“health promotion” [MESH] AND (diversity OR acculturation OR “health services for the aged” [MESH]); (“lifestyle intervention” AND aged [MESH])

The first author (QL) was responsible for the literature search, screened all titles and applied the eligibility criteria in collaboration with the last author (SDI). All authors were however involved and made significant contributions to the identification of relevant publications. When there were uncertainties regarding the relevance after screening of titles, the publications’ abstracts were assessed for relevancy by all authors.

### Data collection

Data from all included publications were extracted using a predefined list based on Cochrane Collaboration’s handbook for systematic reviews [17]. This list included: date of publication, participant data, methods, intervention details, control group details, study quality and results. For the meta-analyses, additional data on treatment effects were extracted for the following outcomes: general health, mental health, physical health, depression, and vitality. In order to be able to calculate standardised mean differences (SMD) for the selected outcomes, data on pre-test and post-test means and standard deviations (SD) were extracted, or calculated when no such data were provided in the publication.

### Evaluation of publication quality

As a marker of study quality a risk of bias assessment was performed with consideration to the following aspects: randomisation (method and concealment), blinding (participants, providers, outcome assessors), dropout rates, intention-to-treat analysis, similarity of baseline characteristics, co-interventions, compliance, timing of outcome assessment [17]. Publications could score a maximum of 12 “yes” responses, and more than six “yes” responses represented a low risk of bias. All three authors were involved in the risk of bias assessment and a consensus method was employed to solve disagreements. Study authors were contacted for additional information when the publications did not contain enough information to assess the risk of bias.

### Qualitative analysis

A narrative synthesis [19, 20] of extracted data on intervention details was undertaken in order to categorise the essential parts of the interventions’ content into so called core components. First, all descriptions of the interventions were analysed together in order to disclose overall features. Second, an iterative process was initiated to categorise and extract the strongest features, progressively sifting out the weakest by a dialectal movement between the data and the emerging categories that represent the core components.

### Statistical analysis

Random effects meta-analyses were performed with Review manager 5.3 [Revman version 5.2.8, Cochrane Collaboration, Oxford, UK] in order to estimate the average effect of health promotion on general health, mental health, physical health, depression, and vitality. SMD between intervention group participants and control group participants were calculated, and the limit for clinical relevance was set at a low level of SMD = 0.12 in order to detect even small effect sizes that can be of importance for individual persons. Statistical heterogeneity was assessed with chi-square and I^2^ statistics. An I^2^ value of 0 % indicates absence of heterogeneity, <25 % indicates low, values between 25 and 50 % moderate, and >50 % high heterogeneity [21].

For evidence grading of the estimated effect on each outcome, the four level system developed by the GRADE working group [22] was used. This system ranges from level one that represents very low quality of evidence and great insecurity of results, to level four representing high quality of evidence and very small insecurity of results [22]. Considering the publications’ RCT design, the grading commenced at the highest level of quality, lowered by one level for each of the following scientific considerations: risk of bias, consistency of results, directness (generalisability), and precision (sufficient data).

## Results

**Fig. 1:**
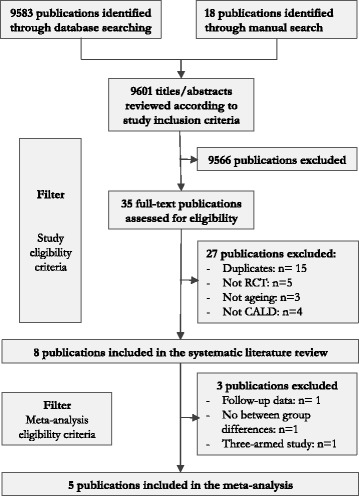
Flowchart. Flowchart over the identification and inclusion of eligible publications

The search process rendered a total of 9601 publications that were screened for eligibility. Following the screening procedure, 20 publications were reviewed in full-text, and finally eight publications that met the eligibility criteria were identified (Fig. 1).

Four of the included publications evaluated occupational therapy programmes with the aim to promote health [23-26], and four evaluated different means of physical activity interventions with the aim to promote health [27-30]. All programmes had community settings with lengths from six weeks up to nine months, and the intensity ranged from three sessions spread out over a seven-week period, up to two hours per week over a nine-month period. A total of 1417 participants were included: 668 were randomised to health promotion and 749 were randomised to control. Women constituted 69 % and men 31 %. The mean age was 65.3 years, ranging from 54.5 to 74.9 years. The participants’ backgrounds were described in different ways: African-American (23 %), white (18 %), Asian (13 %), Hispanic (12 %), immigrants (9 %), Native American (9 %), CALD (8 %), Mandarin-speaking Chinese (4 %), and other (4 %). Six publications were conducted in the United States (*n* = 1170), one in Australia (*n* = 121) and one in the Netherlands (*n* = 126). More detailed information can be found in Additional file 2.

### Publication quality

The risk of bias assessment showed a range from five to nine criteria met, with seven publications having a low risk of bias [23-27, 29, 30] and the remaining [28] having a high risk of bias (Table 1).

**Table 1:** Risk of bias assessment. Assessment of sources of risk of bias within publications

Criteria	References							
	Clark et al. (1997) [23]	Reijneveld et al. (2003) [27]	Sawchuk et al. (2008) [29]	Clark et al. (2001) [24]	Borschmann et al. (2000) [30]	Clark et al. (2012) [26]	Jackson et al. (2000) [25]	Resnick et al. (2008) [28]
1. Adequate method of randomisation?	Y	Y	Y	Y	Y	Y	Y	Y
2. Allocation concealment?	Y	Y	Y	Y	Y	U	Y	U
3. Patient blinding?	N	N	N	N	Y	N	N	N
4. Provider blinding?	N	N	N	N	N	N	N	N
5. Outcome assessor blinding?	Y	Y	N	Y	Y	Y	Y	U
6. Dropout rate described and acceptable?	Y	N	Y	N	N	N	N	N
7. All participants analysed in allocated group?	Y	Y	Y	Y	U	Y	U	U
8. Free of suggestive/selective outcome reporting?	Y	Y	Y	Y	N	Y	Y	Y
9. Similarity of baseline characteristics?	Y	Y	Y	Y	Y	Y	Y	Y
10. Co-interventions avoided or similar?	U	U	U	U	U	U	U	U
11. Compliance acceptable?	Y	Y	Y	Y	Y	Y	Y	Y
12. Timing of outcome assessment similar?	Y	Y	Y	Y	Y	Y	Y	Y
Total 0–12 Y	9	8	8	8	7	7	7	5

*Y* Yes, *N* No, *U* Unsure

More than six Y = Low risk of bias

### Narrative synthesis–core components of the health promotion programmes

The narrative synthesis resulted in the following core components: activity, cultural and linguistic modifications, a person-centred approach, health information, and professional provision (Table 2).

**Table 2:** Core components. Core components of the health promotion programmes

Author, year [reference]	Activity	Cultural and linguistic modifications	A person-centred approach	Health information	Professional provision
Borschmann et al. 2010 [30]	Physical activities	Yes	Yes	No	Yes
Clark et al. 1997 [23] Clark et al. 2001 [24] Clark et al. 2012 [26]	Meaningful activities	Yes	Yes	Yes	Yes
Jackson et al. 2000 [25]	Meaningful activities	Yes	Yes	Yes	Yes
Reijneveld et al. 2003 [27]	Physical activities	Yes	No	Yes	No
Resnik et al. 2002 [28]	Physical activities	Yes	Yes	Yes	Yes
Sawchuck et al. 2008 [29]	Physical activities	No	No	Yes	No

#### Activity

All programmes included activity, albeit applied in different ways. Predominantly, the programmes had a physical approach to health promotion, with focus on: resistance exercises and stretching with instructions in lay language [28], weekly physical activity monitoring [29], low intensity exercises [27], or discussions on how to overcome individual barriers to physical activities [30]. The aim of those programmes was to increase the participants’ physical activity levels to improve their health. The remaining programmes [23-26] had an occupational science approach, which means that they aimed to provide support on how health could be promoted by meaningful daily activities.

#### Cultural and linguistic modifications

Five programmes involved modifications in relation to the participants’ cultural and linguistic affiliations. Linguistic modifications involved the use of interpreters [30], providers who could communicate with the participants in their mother tongue [23-26] and translated written material [25, 27, 28]. Three programmes made cultural modifications to the health information [25, 27, 28], and two to the physical exercises according to music, dance, and instructions [27, 28]. One programme employed the use of a peer educator from the same country as the participants when possible, and divided participants by gender according to their choice [27]. Another programme took specific consideration to cultural differences in interpersonal dynamics and norms regarding presentation of information [25]. The participants in this programme were also provided with instructions on how to deal with everyday mainstream culture issues in the society.

#### A person-centred approach

With focus on personal goal setting and interactive group settings four programmes [23-26, 28, 30] were considered to have a person-centred approach. Employing peer support, collective- and self-efficacy, those programmes’ content took a step away from objectification, and instead regarded the participants as persons with unique experiences and needs. Through personal goal setting, the participants were strengthened in their capabilities to implement meaningful lifestyle changes. They received advice and support in their establishment of personal goals, and each person’s plan was followed up throughout the programme. Interactive group settings and peer support used collective efficacy to effectively direct the participants’ activities towards the achievement of their personal goals. With regard to self-efficacy, one programme [28] employed different means of strengthening the participants’ confidence in their own ability and motivation for change.

#### Health information

Five programmes [23-29] included health information in their content to inform participants on: nutrition, benefits of physical exercise, knowledge on how to select or perform daily activities, safety in and around the participant’s home, symptoms related to ageing, and how to achieve and maintain a healthy and satisfying lifestyle. The health information was provided in group settings, by occupational therapists in two of the programmes [23-26], and by a peer educator in one of the programmes [27]. In the two remaining programmes the information was provided individually through written educational materials [28], or by written education materials in combination with oral information from a research assistant [29].

#### Professional provision

Four of the programmes were professionally provided. Two of the programmes were provided by registered occupational therapists [23-26], one by an exercise physiologist [30], and one by a dietician [28]. The programmes delivered by occupational therapists also employed a programme specific education, and regular meetings were described to have been held between the providers and the on-site project director and manager to secure a professional provision of one of those programmes [26].

### Meta-analyses of the health promotion programmes’ effect

Data on outcomes reported in at least three publications were analysed in order to estimate an average effect of health promotion on a variety of health related outcomes among ageing persons with CALD backgrounds.

#### General health

Results on general health from 566 participants in three publications [23, 25, 26] were entered into meta-analysis, revealing a pooled effect on the rim of statistical significance (SMD = 0.17, 95 % CI = −0.00 to 0.34). This effect was considered clinically relevant, but considering the elevated heterogeneity (I^2^ = 58 %, *p* = 0.05), and limited scientific foundation (GRADE level 2, low quality of evidence) there is more research needed in order to secure the estimations. For more detailed information and forest plot see Table 3.

**Table 3:**
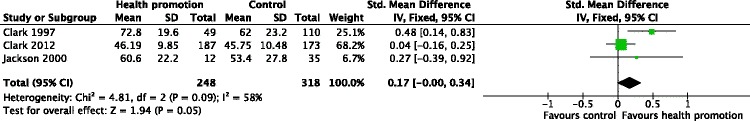
General health. General health post-treatment, health promotion programmes versus control (results from 3 publications)

#### Mental health

Data from 760 participants in five publicat ions [23-28] were pooled in meta-analysis, rendering a statistically significant and clinically relevant pooled effect on improved mental health in the intervention group (SMD = 0.55, 95 % CI = 0.17 to 0.92). The test for heterogeneity did however uncover high heterogeneity (I^2^ 80 %, *p* = 0.004), invalidating the findings. In addition, the scientific foundation is limited (GRADE level 2, low quality of evidence). For detailed information and forest plot, see Table 4.

**Table 4:**
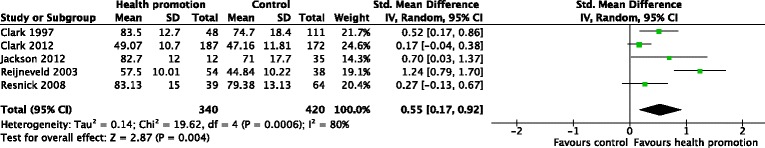
Mental health. Mental health post-treatment, heatlh promotion programmes versus control (results from 5 publications)

#### Physical health

Five publications [23-28] evaluated the effect of health promotion on physical health, and data from 790 participants were pooled in a meta-analysis that rendered a statistically significant and clinically relevant pooled effect in favour for health promotion (SMD = 0.26, 95 % CI = 0.04 to 0.49). With moderate heterogeneity (I^2^ 50 %, *p* = 0.02), and low quality of evidence (GRADE level 2), the scientific foundation for the effect of health promotion on physical health is however limited. For detailed information and forest plot, see Table 5.

**Table 5:**
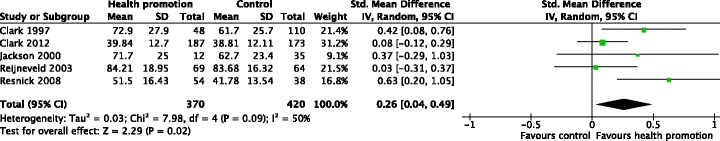
Physical health. Physical health post-treatment, health promotion programmes versus control (results from 5 publications)

#### Depression

Data from three publications [23, 26, 28] comprising 766 participants were pooled for the effect size of depression (Table 6). The meta-analysis revealed low heterogeneity (I^2^ 12 %, *p* = 0.007), suggesting good prospects for the average pooled estimates of a significantly lower risk for depression in the intervention group (SMD = −0.22, 95 % CI = −0.38 to −0.06). Those effects were considered clinically relevant, indicating that health promotion is superior to usual care or a social intervention for depression, but the scientific foundation is limited (GRADE level 2, low quality of evidence).

**Table 6:**
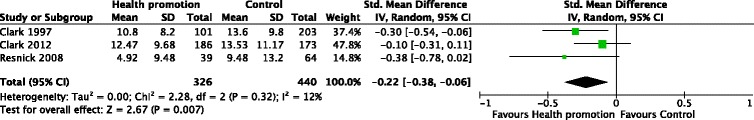
Depression. Depression post-treatment, health promotion programmes versus control (results from 3 publications). A lower value indicates improvement for this outcome, which is why health promotion is presented to the left

#### Vitality

The pooled effect on vitality from three publications [23, 26, 28] with 565 participants was just above statistical significance in favour for health promotion (SMD = 0.30, 95 % CI = 0.01 to 0.59). The effect was clinically relevant, but the moderate heterogeneity (I^2^ = 49 %, *p* = 0.04), and limited scientific foundation (GRADE level 2, low quality of evidence) indicate that there is more research needed to secure the effect of health promotion on vitality. For more detailed information and forest plot see Table 7.

**Table 7:**
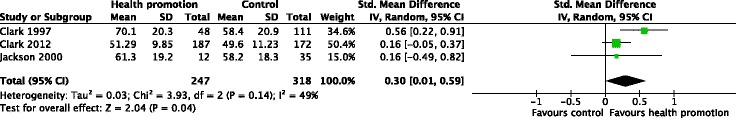
Vitality. Vitality post-treatment, heatlh promotion programmes versus control (results from 3 publications)

## Discussion

This study provides a unique mapping of the content and effects of multidimensional health promotion programmes that have included ageing persons with CALD backgrounds. Visualising five core components, the findings of the review suggest a multidimensional health promotion design, and those findings are strengthened by the estimated effects on both mental and physical health revealed in the meta-analyses. Concurrent with WHO statements [31], and previous research on health promotion with older people [4, 32], the combined findings of this study encourage the inclusion of activity components and health information in health promotion programmes. What the present findings add to the scientific knowledge is a visualisation of how those two components might need to be culturally and linguistically adapted to suit the needs of an increasingly diverse ageing population. The presentation of means to bridge cultural and linguistic barriers between healthcare providers and older people with CALD backgrounds provides important information on how to make health promotion more accessible for this part of the population. However, the included publications also acknowledge hetereogeneity within CALD populations, and the findings support a person-centred approach to health promotion, with attention to each person’s preferences and needs. As described by Turner et al. [33], person-centredness is an important part of best practice with ageing persons [33], and it is an important aspect of confronting stereotypic and stigmatising views of people with CALD backgrounds [34, 35]. Nevertheless, coming to a fore is a scientific knowledge gap with regard to how a person-centred approach could be applied to health promotion with older people with CALD backgrounds. As visualised by Hussain-Gambles et al. [36], a major problem is that researchers tend to exclude people with different cultural, linguistic, ethnic or national backgrounds in clinical trials. The present review confirms this finding, and encourages future research to put focus on the operationalisation of person-centredness into health promotion actions with an ageing and increasingly diverse population.

### Methodological limitations

In the light of the present findings presented and discussed above, there are some concerns regarding the amount and quality of identified publications, as well as the heterogeneity of their participants. Therefore, the findings should be interpreted with caution, and in the light of several limitations.

First, the sparse amount of identified publications urges a questioning of the narrow inclusion criteria. The exclusion of publications on disease prevention did minimise the amount of eligible publications significantly. This was, however, considered relevant based on previous findings, which suggests that neither disease prevention nor promotion of a physically active lifestyle alone is enough to promote such a complex process as health over the ageing process [2, 3]. The limitation of designs to only include RCT publications also narrowed down the amount of eligible publications significantly. In return, the precision of identified findings was enhanced, and the possibility to provide evidence for the programmes’ efficacy improved.

Second, the majority of the reviewed trials were conducted in the United States, which makes the transferability and global application of the findings questionable. The findings show that there are commonalities across different programmes in different contexts, and that there is some scientific foundation for their efficacy. Nevertheless, consideration always needs to be given to the local contexts in which health promotion programmes ought to be implemented, and with attention to the heterogeneity of persons as well as different groups of people. Even though the meta-analyses demonstrate statistically significant effects in favour of health promotion, it is important to remember that there is no guarantee for efficacy or success in different contexts or with different populations.

Third, because data were not consistent in the publications included in the meta-analyses, it is impossible to explain to what extent health promotion programmes could contribute to improved health for older people with CALD backgrounds. In addition, the analysed interventions were carried out on small populations with heterogeneous backgrounds, and the identified publications were clinically heterogeneous. Therefore, a randomised effects model for meta-analysis was applied in order to minimise the effect of clinical heterogeneity. However, there is always a risk of drawing too generalised conclusions from a pooling of results, and the dearth of high quality RCT publications makes the evidence for the programmes’ efficacy low. Where evidence is available, publications are subject to a number of methodological limitations that cloud the conclusions arising from them.

## Conclusions

This study provides a unique mapping of the content and effects of health promotion programmes for ageing persons with culturally and linguistically diverse backgrounds. One of the major findings is the visualisation of how understudied this particular research field actually is. It is well known that ageing persons benefit from multidimensional health promotion programmes commenced before the onset of disease, and the visualised dearth of research with regard to ageing persons with culturally and linguistically diverse backgrounds thus poses serious threats to health equity. More research is needed in order to fully explore how to promote the health of an ageing and increasingly diverse population, and a randomised controlled trial design is suggested in order to provide ample evidence for health promotion programme efficacy.

## Additional files

Additional file 1:
**PRISMA checklist.** The PRISMA checklist used for the manuscript preparation. (PDF 358 kb) Additional file 2:
**Data extraction form.** Data extraction form. (DOC 91 kb)

## Abbreviations

CALD: Culturally and linguistically diverse
CERAD: Consortium to establish a registry of alzheimer’s disease
CES-D: Center for epidemiologic studies depression scale
CHAMPS: Community healthy activities program for seniors questionnaire
CI: Confidence interval
FSQ: Functional status questionnaire
GDS: Geriatric depression scale
HAP-AAS: Human activity profile adjusted activity score
LSI-Z: Life satisfaction index-z
MHI-5: Mental health index (from SF-36)
MOS: Medical outcomes study
NRS: Numeric rating scale
OEE: Outcome expectations for exercise scale
SEE: Self-efficacy for exercise scale
SF-12: RAND short form-12
SF-36: RAND short form-36
SMD: Standardised mean difference
SOC-13: Stages of change questionnaire
WHO: World Health Organization
YPAS: Yale physical activity survey
