# Supplementary material for: Health promotion programme design and efficacy in relation to ageing persons with culturally and linguistically diverse backgrounds: a systematic literature review and meta-analysis
Source: BMC Health Serv Res. 2015 Dec 16;15:560. doi: 10.1186/s12913-015-1222-4 (PMC4682220; doi:10.1186/s12913-015-1222-4)
Supplement: Additional file 2: — Data extraction form. Data extraction form. (DOC 91 kb) [file 12913_2015_1222_MOESM2_ESM.doc]

**Additional file 2.** Data extraction form

| **Author, year, reference, country** | **Participants and methods** | **Intervention group details** | **Control group details** | **Outcome results (p-values)***  Significance level α 0.05 | **Study quality**  **Comments** |
| --- | --- | --- | --- | --- | --- |
| Borschmann et al. 2010 [30]  Australia | **Participants**  Culturally and linguistically diverse older adults (Macedonian 75%, Polish 25%)  Mean age 69,9 years  63% female  **Eligibility criteria**   - ≥55 years - Able to engage in discussion - Independently ambulant for at least 5 minutes, with or without walking aid   **Setting**  Community setting  **Follow-up**  Immediately after completion of sessions | **Programme**  Three one-hour group sessions over a 7-week period to discuss motivation for and barriers to physical activity. An interpreter was used for all components of the intervention.  **Providers**  Exercise physiologist  **No of participants**  61 at baseline and 48 at follow-up | **Type of control**  A one-hour fall prevention talk on nutrition, environmental modification, medical check-ups, brief mention of physical activities  **Providers**  Exercise physiologist  **No of participants**  60 at baseline and 45 at follow-up | **Favours control**   - Preparation to undertake physical activity (SoCQ)   **Favours none**   - Steps per week (pedometers) - Self-reported functional activity ability (HAP-AAS) - Self-rated health (SF-36) - Mobility (6-minute walk test) - Perceived exertion (Borg perceived exertion scale) - Gait velocity and stride length (6-minute walk test) - Global leg strength (5-times-sit-to-stand test) - Total body fat estimation (Body Mass index)   No outcome data was reported on any of the outcome measures. | **Risk of bias assessment:**  7/12  **Intention to treat analysis:** No  **Comments:** Few participants were able to provide valid responses to the Borg scale. The lack of between group differences could be explained by random error, sample size or ineffective intervention. |

**Additional file 2.** Data extraction form

| **Author, year, reference, country** | **Participants and methods** | **Intervention group details** | **Control group details** | **Outcome results (p-values)***  Significance level α 0.05 | **Study quality**  **Comments** |
| --- | --- | --- | --- | --- | --- |
| Clark et al. 1997 [23]  United States | **Participants**  Culturally diverse older adults (Asian 47 %, white 23 %, African-American 17 %, Hispanic: 11 %, other 2%)  Mean age 74,4 years  65% female  **Eligibility criteria**   - ≥60 years - Independent living, culturally diverse - Capacity to benefit from occupational therapy   Excluding people unable to live independently or with signs of dementia  **Setting**  Community setting  **Follow-up**  Immediately after completion of sessions | **Programme**  Two hours of occupational therapy in group per week and a total of nine hours of individual occupational therapy over a nine month period. Linguistically modified health information.  **Providers**  Occupational therapists  **No of participants**  122 at baseline  48-102 at follow-up (depending on outcome measure) | **Type of control**  A social control programme or no intervention (pooled by study authors due to homogeneity at baseline and follow-up)  **Provider**  Nonprofessional  **No of participants**  239 at baseline  110-202 at follow-up (depending on outcome measure) | **Favours health promotion**   - Instrumental activities of daily living (FSQ) p=0.28 - Social activities (FSQ) p=0.38 - Quality of interaction (FSQ) p=0.03 - Life satisfaction (LSI-Z) p=0.03 - Depression (CES-D) p=0.16 - Health perception (MOS) p=0.05 - Bodily pain (SF-36) p=0.03 - Physical functioning (SF-36) p=0.008 - Role functioning (SF-36) p=0.02 - General health (SF-36) p=0.06 - Vitality (SF-36) p=0.004 - Social functioning (SF-36) p=0.05 - Emotional role functioning (SF-36) p=0.05 - Mental health (SF-36) p=0.02)   **Favours control**   - Basic activities of daily living (FSQ) p=0.31 | **Risk of bias assessment:** 9/12  **Intention to treat analysis:** Yes |

**Additional file 2.** Data extraction form

| **Author, year, reference, country** | **Participants and methods** | **Intervention group details** | **Control group details** | **Outcome results (p-values)**  Significance level α 0.05 | **Study quality**  **Comments** |
| --- | --- | --- | --- | --- | --- |
| Clark et al. 2001 [24]  United States | Same population, eligibility criteria and setting as Clark et al 1997 [23]  **Follow-up**  6 months after intervention completion | Same programme and providers as Clark et al 1997 [23]  **No of participants**  122 at baseline  47-96 at follow-up (depending on outcome measure) | Same control and providers as Clark et al 1997 [23]  **No of participants**  239 at baseline  104-189 at follow-up (depending on outcome measure) | **Favours health promotion**   - Quality of interaction (FSQ) p=0.05 - Life satisfaction (LSI-Z) p=0.23 - Health perception (MOS) p=0.45 - Bodily pain (SF-36) p=0.08 - Physical functioning (SF-36) p=0.03 - Role functioning (SF-36) p=0.02 - General health (SF-36) p=0.06 - Vitality (SF-36) p=0.001 - Social functioning (SF-36) p=0.01 - Emotional role functioning (SF-36) p=0.05 - General mental health (SF-36) p=0.02   **Favours control**   - Basic activities of daily living (FSQ) p=0.17 - Instrumental activities of daily living (FSQ) p=0.49 - Social activities (FSQ) p=0.49 - Depression (CES-D) p=0.20 | **Risk of bias assessment:** 8/12  **Intention to treat analysis:** Yes  **Comments:**  Long-term follow-up of Clark et al 1997 [23] |

**Additional file 2.** Data extraction form

| **Author, year, reference, country** | **Participants and methods** | **Intervention group details** | **Control group details** | **Outcome results (p-values)**  Significance level α 0.05 | **Study quality**  **Comments** |
| --- | --- | --- | --- | --- | --- |
| Clark et al. 2012  [26]  United States | **Participants**  Ethnically diverse older people (White 37%, Black/African-American: 34%, Hispanic or Latino 21%, Asian 4%, other 4%)  Mean age 74,9 years  66% female  **Eligibility criteria**   - ≥60 years - Residents/users of study recruitment sites - No overt signs of psychosis or dementia - Able to complete the study assessment battery (with assistance, if necessary)   **Setting**  Community setting  **Follow-up**  Immediately after intervention completion | **Programme**  Weekly 2-hour occupational therapy sessions in groups, and up to 10 hours of individual 1-hour occupational therapy sessions over a 6-month period  **Providers**  Registered occupational therapists  **No of participants**  232 at baseline  166-187 at follow-up (depending on outcome measure) | **Type of control**  No treatment  **Providers**  Not applicable  **No of participants**  228 at baseline  152-173 at follow-up (depending on outcome measure) | **Favours health promotion**   - Physical functioning (SF-36) p=0.09 - Role functioning (SF-36) p=0.18 - Bodily pain (SF-36) p=0.02 - General health (SF-36) p=0.25 - Vitality (SF-36) p=0.03 - Social function (SF-36) p=0.04 - Emotional role functioning (SF-36) p=0.16 - Mental health (SF-36) p=0.03 - Physical composite (SF-36) p=0.09 - Mental composite (SF-36) p=0.03 - Life satisfaction (LSI-Z) p=0.03 - Depression (CES-D) p=0.03 - Memory, delayed recall (CERAD-memory) p=0.38 - Memory, recognition (CERAD-memory) p=0.26 - Visual search p=0.49 - Psychomotor speed p=0.49   **Favours control**   - Memory, immediate recall (CERAD-memory) p=0.2 | **Risk of bias assessment:** 7/12  **Intention to treat analysis:** Yes  **Comments:**  Spanish versions of the questionnaires were provided to 68 participants |

**Additional file 2.** Data extraction form

| **Author, year, reference, country** | **Participants and methods** | **Intervention group details** | **Control group details** | **Outcome results (p-values)**  Significance level α 0.05 | **Study quality**  **Comments** |
| --- | --- | --- | --- | --- | --- |
| Jackson et al. 2000  [25]  United States | **Participants**  Two subgroups from the study population in Clark et al 1997 [30]:   - Mandarin-speaking Chinese men and women - English-speaking men and women   Mean age 73,9 years  73% female  **Eligibility criteria** Same as Clark et al 1997 [30]  **Setting**  Community setting  **Follow-up**  Immediately after completion of sessions | **Programme**  A culturally and linguistically adapted version of the programme described by Clark et al 1997 [30].  **Providers**  Occupational therapists  **No of participants**  Mandarin-speaking: 16 at baseline, 12 at follow-up  English-speaking: 38 at baseline, 29 at follow-up | Same control and providers as Clark et al 1997 [31]  **No of participants**  Mandarin-speaking: 42 at baseline, 35 at follow-up  No English-speaking control group | **Favours health promotion:**   - Bodily pain (SF-36) p=0.22 - Physical functioning (SF-36) p=0.14 - Role functioning (SF-36) p=0.09 - General health (SF-36) p=0.20 - Vitality (SF-36) p=0.21 - Social functioning (SF-36) p=0.26 - Emotional role functioning (SF-36) p=0.13 - Mental health (SF-36) p=0.10   Outcome results are reported on comparisons within the Mandarin-speaking cohort (intervention vs control). | **Risk of bias**  7/12  **Intention to treat analysis:** No |

**Additional file 2.** Data extraction form

| **Author, year, reference, country** | **Participants and methods** | **Intervention group details** | **Control group details** | **Outcome results (p-values)**  Significance level α 0.05 | **Study quality**  **Comments** |
| --- | --- | --- | --- | --- | --- |
| Reijneveld et al. 2002  [27]  The Netherlands | **Participants**  Elderly Turkish immigrants  Mean age 54,5 years  75% female  **Eligibility criteria**   - Born in Turkey - ≥45 years   **Setting** Community setting  **Follow-up**  Immediately after completion of sessions | **Programme**  Six 2-hour sessions of culturally and language adapted health information and low intensity exercises in groups  **Providers**  Peer educator Exercise instructor  **No of participants**  74 at baseline  54 at follow-up | **Type of control**  Available welfare services for the elderly  **Providers**  Healthcare  professionals when needed  **No of participants**  52 at baseline  38 at follow-up | **Favours health promotion**   - Physical well-being (SF-12) p=0.78 - Mental well-being (SF-12) p=0.08 - Mental health (MHI-5 scale of SF-36) p=0.03   **Favours control**   - Health knowledge (shortened Voorrips questionnaire) p=0.78 - Physical activity level p=0.76 | **Risk of bias assessment:** 8/12  **Intention to treat analysis:** Yes  **Comments:**  There were differences reported on mental well-being by age group, with effects larger for participants aged 55 years or older. |

**Additional file 2.** Data extraction form

| **Author, year, reference, country** | **Participants and methods** | **Intervention group details** | **Control group details** | **Outcome results (p-values)**  Significance level α 0.05 | **Study quality**  **Comments** |
| --- | --- | --- | --- | --- | --- |
| Resnick et al. 2008  [28]  United States | **Participants**  Minority older adults  (African-American: 73 %, Latino: 20 %, other: 7 %)  Mean age 73,3 years  81% female  **Eligibility criteria**   - ≥60 years - Blood pressure 200/100 - Heart-rate 60-120 - No known recent history of heart attack, stroke or new irregular heartbeat - Acknowledgment of participation from the person’s primary healthcare provider   **Setting**  Community setting  **Follow-up**  2-4 weeks after completion of sessions | **Programme**  Culturally adapted physical exercises, linguistically adapted health information, and an efficacy-enhancement component in a group setting, reviewing the benefits of exercise and physical activity and ways to overcome barriers. The intervention took place twice a week over a 12 week period  **Providers**  Lay exercise trainers  Dietician  **No of participants**  100 at baseline  64 at follow-up | **Type of control**  Routine care and activities  **Providers**  Healthcare  professionals when needed  **No of participants**  66 at baseline  39 at follow-up | **Favours health promotion**   - Self-efficacy (SEE scale) p=0.21 - Outcome expectations related to exercise (OEE scale) p=0.02 - Time spent in exercise (YPAS) p=0.04 - Time spent in overall activities (YPAS) p=0.63 - Depression (GDS) p=0.02 - Chair rise p=0.05 - Mobility (Tinetti scale) p=0.85 - Mental health (SF-12) p=0.22   **Favours control**   - Pain (NRS) p=0.35 - Fear of falling p=0.17 - Physical health (SF-12) p=0.85 | **Risk of bias**  5/12  **Intention to treat analysis:** No  **Comment:**  The high risk of bias mostly considered criteria that were not described or non applicable (i.e blinding of participants and providers) |

**Additional file 2.** Data extraction form

| **Author, year, reference, country** | **Participants and methods** | **Intervention group details** | **Control group details** | **Outcome results (p-values)**  Significance level α 0.05 | **Study quality**  **Comments** |
| --- | --- | --- | --- | --- | --- |
| Sawchuck et al. 2008  [29]  United States | **Participants**  Native elders  Mean age 58 years  74,5% female  **Eligibility criteria**   - 50-74 years - Having a sedentary lifestyle - Being able to walk without assistance - Denying medical contraindications to walking - Living within a 2 hour drive from the study setting   **Setting**  Community setting  **Follow-up**  Immediately after completion of sessions | **Programme**  Weekly activity monitoring with pedometer, and a booklet for self-monitoring of physical activities. Participants also completed two clinic visits and a phone call to bolster participation, boost engagement in physical activities, and address concerns regarding the study. All participants also received an educational leaflet on the health benefits of increased physical activity  **Providers**  Research assistant  **No of participants**  63 at baseline  58-61 at follow-up (depending on outcome measure) | **Type of control**  Same content, intensity and provider as the health-promotion programme, excluding the use of pedometers  **No of participants**  62 at baseline  53-56 at follow-up (depending on outcome measure) | **Favours health promotion with pedometer**   - Emotional role functioning (SF-36) p=0.47 - Social functioning (SF-36) p=0.24 - Mental health (SF-36) p=0.51 - Vitality (SF-36) p=0.88 - General health (SF-36) p=0.32 - Mental composite (SF-36) p=0.14   **Favours health promotion with activity monitoring**   - Kcal expended/week in all physical exercises (CHAMPS) p=0.27 - Kcal expended/week in moderate-intensity physical exercises (CHAMPS) p=0.19 - Frequency of all exercise-related activities p=0.65 - Frequency of moderate-intensity exercise-related activities p=0.82 - Physical functioning (SF-36) p=0.83 - Role functioning (SF-36) p=0.15 - Bodily pain (SF-36) p=0.32 - Physical composite (SF-36) p=0.13 | **Risk of bias**  8/12  **Intention to treat analysis:** Yes  **Comment:**  The lack of between-group effects might have been a result of the two study arms being too similar. |
